# Cumulative Evidence for Relationships Between 8q24 Variants and Prostate Cancer

**DOI:** 10.3389/fphys.2018.00915

**Published:** 2018-07-16

**Authors:** Yu Tong, Tao Yu, Shiping Li, Fengyan Zhao, Junjie Ying, Yi Qu, Dezhi Mu

**Affiliations:** ^1^Department of Pediatrics, West China Second University Hospital, Sichuan University, Chengdu, China; ^2^Key Laboratory of Obstetric & Gynecologic and Pediatric Diseases and Birth Defects of Ministry of Education, West China Second University Hospital, Sichuan University, Chengdu, China

**Keywords:** 8q24, genetic variant, prostate cancer, susceptibility, meta-analysis

## Abstract

Multiple independent cancer susceptibility loci at chromosome 8q24 have been identified by GWAS (Genome-wide association studies). Forty six articles including 60,293 cases and 62,971 controls were collected to conduct a meta-analysis to evaluate the associations between 21 variants in 8q24 and prostate cancer risk. Of the 21 variants located in 8q2\5 were significantly associated with the risk of prostate cancer. In particular, both homozygous AA and heterozygous CA genotypes of rs16901979, as well as the AA and CA genotypes of rs1447295, were associated with the risk of prostate cancer. Our study showed that variants in the 8q24 region are associated with prostate cancer risk in this large-scale research synopsis and meta-analysis. Further studies are needed to explore the role of the 8q24 variants involved in the etiology of prostate cancer.

## Introduction

Prostate cancer (PCa) is the commonest non-cutaneous malignancy in men all over the world. Based on epidemiological and biological data, there is growing evidence that many influencing factors, including geography, ethnicity, genetic factors, and so on(Rebbeck, 2017), are associated with the risk of PCa. PCa exhibits high heritability, however, the exact etiology of PCa is still unknown. Identification of genetic factors regulating the susceptibility and progression of PCa contributes to improvement of preventive measures and therapeutic outcomes.

Multiple risk loci for prostate cancer have been identified by GWAS. In 2007, a two-stage GWAS from 1,854 prostate cancer patients and 1,894 population-screened controls was conducted. In this study, common loci at 8q24 were identified to be associated with prostate cancer (Eeles et al., 2008). It was proved that 8q24 region was associated with lots of cancers, including breast (Pereira et al., 2016), prostate (Hubbard et al., 2016), bladder (Kiltie, 2010), colon (Ling et al., 2013), lung (Zhang et al., 2012), gliomas (Rice et al., 2013), and so on. These susceptibility loci actually do not affect coding DNA, interestingly, these loci showed strong linkage disequilibrium (LD) as they often tightly linked with many SNPs. However, further study found that there are many enhancers in 8q24 region, and the rs6983267-containing enhancer interacts with the MYC gene by binding with TCF7L2 (TCF4), and alter the sensitivity to WNT signaling (Tuupanen et al., 2009). Another recent study found that the rs378854-containing region can interact with the promoters of both MYC and MYC activator PVT1(Meyer et al., 2011). Based on the above compelling evidence, it was supposed that the 8q24 variants played important roles in prostate carcinogenesis.

Here we performed a comprehensive meta-analysis, involving a total of 60,293 cases and 62,971 controls, to evaluate all genetic studies that investigated associations between 15 variants in 8q24 and risk of prostate cancer.

## Methods

### Search strategy and selection criteria

We systematically searched PubMed and Embase to identify genetic association studies published in print or online before January 10th, 2018 in English language using key terms “8q24” and “polymorphism or variant or genotype” and “prostate carcinoma or prostate tumor or prostate cancer”. Two investigators (Yu Tong and Tao Yu) independently assessed the eligibility of each study. All studies included in this meta-analysis must meet all the following inclusion criteria: (i) evaluating the associations of the 8q24 variants with prostate cancer risk; (ii) providing sufficient data or multivariate-adjusted risk estimates [e.g., odds ratios (ORs), hazard ratios (HRs), relative risks (RRs), 95% confidence intervals (CIs) or standard errors (SEs)] to calculate these estimates. The exclusion criteria were as follows: (i) insufficient data; (ii) they were published as letters to editors or conference abstracts; (iii) they were studies about cancer mortality.

### Data extraction

Guidelines recommended were used to report meta-analyses of observational studies by an investigator (Yu Tong and Tao Yu) to extract data. Extracted data efrom each eligible study included name of first author, study design, publication date, source population, ethnicity, sample size, variants, alleles, and genotype counts, Hardy-Weinberg equilibrium (HWE) among controls. Ethnicity was classified as Caucasian, African, Asian, or others such as Latinos and Hawaiians. In this meta-analysis, 46 eligible publications are available with sufficient data.

### Statistical analysis and assessment of cumulative evidence

For each study, the odds ratio (OR) was used as the metric of choice. Pooled odds ratios were computed by the fixed effects model and the random effects model based on heterogeneity estimates, according to Prof. Michael Borenstein's suggestion (Borenstein et al., 2010). Once an overall gene effect was confirmed, the genetic model-free approach suggested by Minelli et al. (2005) was used to estimate the genetic effects and mode of inheritance. Assessment of protection from bias also considered the magnitude of association. OR less than 1.15 implicated presence of bias, unless the association had been replicated prospectivelywith no evidence of publication bias by several studies, such as GWAS or GWAS meta-analysis from collaborative studies. Heterogeneity between studies was evaluated by Cochran's Q test and calculated *I*^2^ statistic h. *I*^2^-values < 25%, 25–50%, and > 50% represent no or little heterogeneity, moderate heterogeneity, and large heterogeneity, respectively. Sensitivity analyses were conducted to examine if exclusion of first published study deviated from HWE in controls influence the significant association. Harbord's test was performed to evaluate publication bias. Small study bias was calculated by egger's test. All analyses were conducted using Stata, version 14.0 (StataCorp, 2017), with the *metan, metabias* commands.

## Results

### Eligible studies

**Figure 1:**
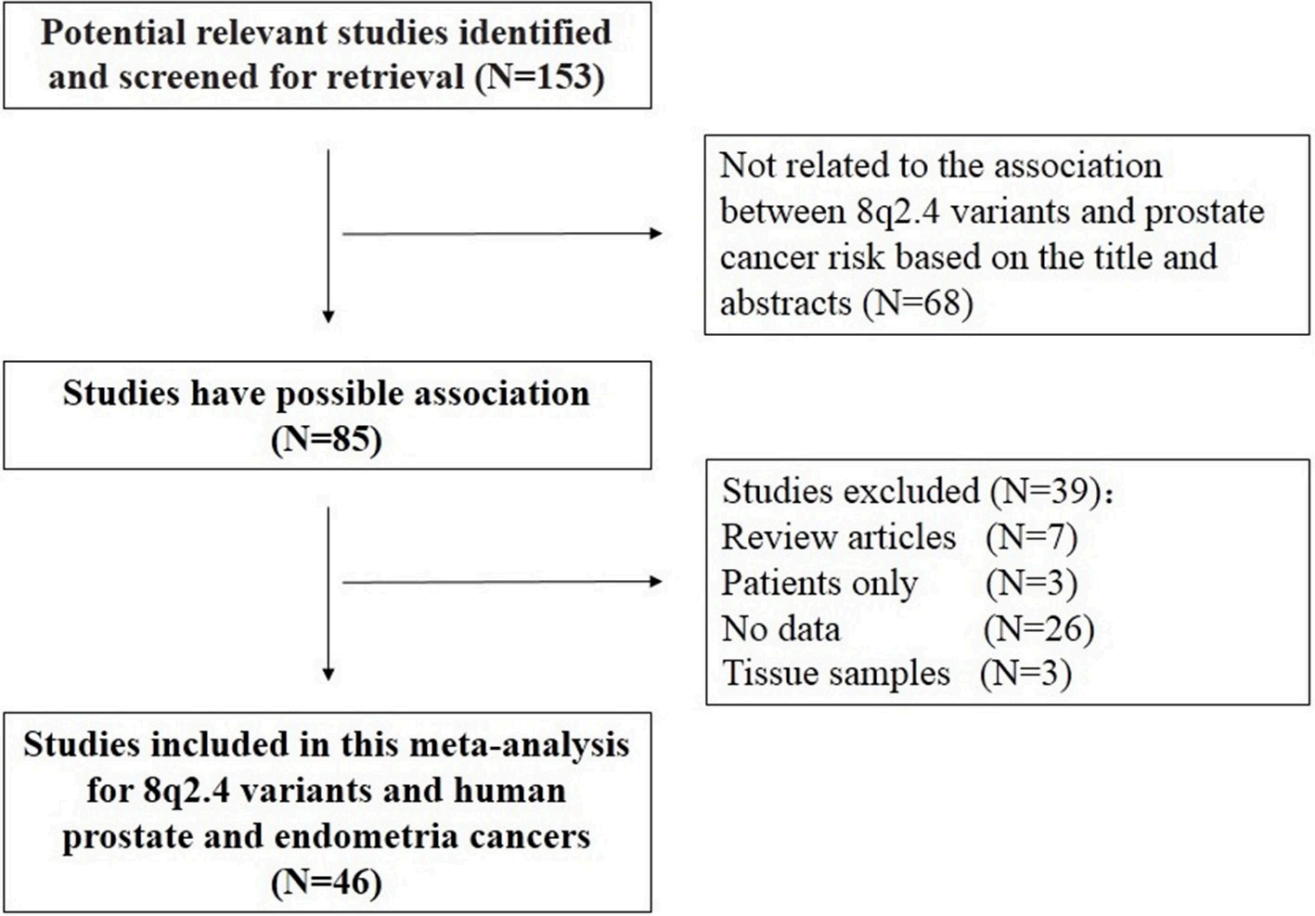
Flow diagram of included and excluded studies.

Our initial database search identified 268 potentially relevant studies. Based on a review of titles and abstracts, 85 articles were retained. The full text of these 85 articles was reviewed in detail, and 46 studies were eligible in this meta-analysis. The specific process for identifying eligible studies and inclusion and exclusion criteria are summarized in Figure 1.

### Allelic associations

Of the 21 variants located in 8q24, 15 were significantly associated with the risk of prostate cancer, including rs16901979, rs1447295, rs6983561, rs7000448, rs6983267, rs13254738, rs7017300, rs7837688, rs1016343, rs7008482, rs4242384, rs620861, rs10086908, DG8S737 Allele−8, and rs10090154. No significant associations were found between rs4242382, rs4645959, rs7837328, rs16901966, rs10505476, rs13281615 and prostate cancer (data not shown).

### rs16901979 C>A

**Figure 2:**
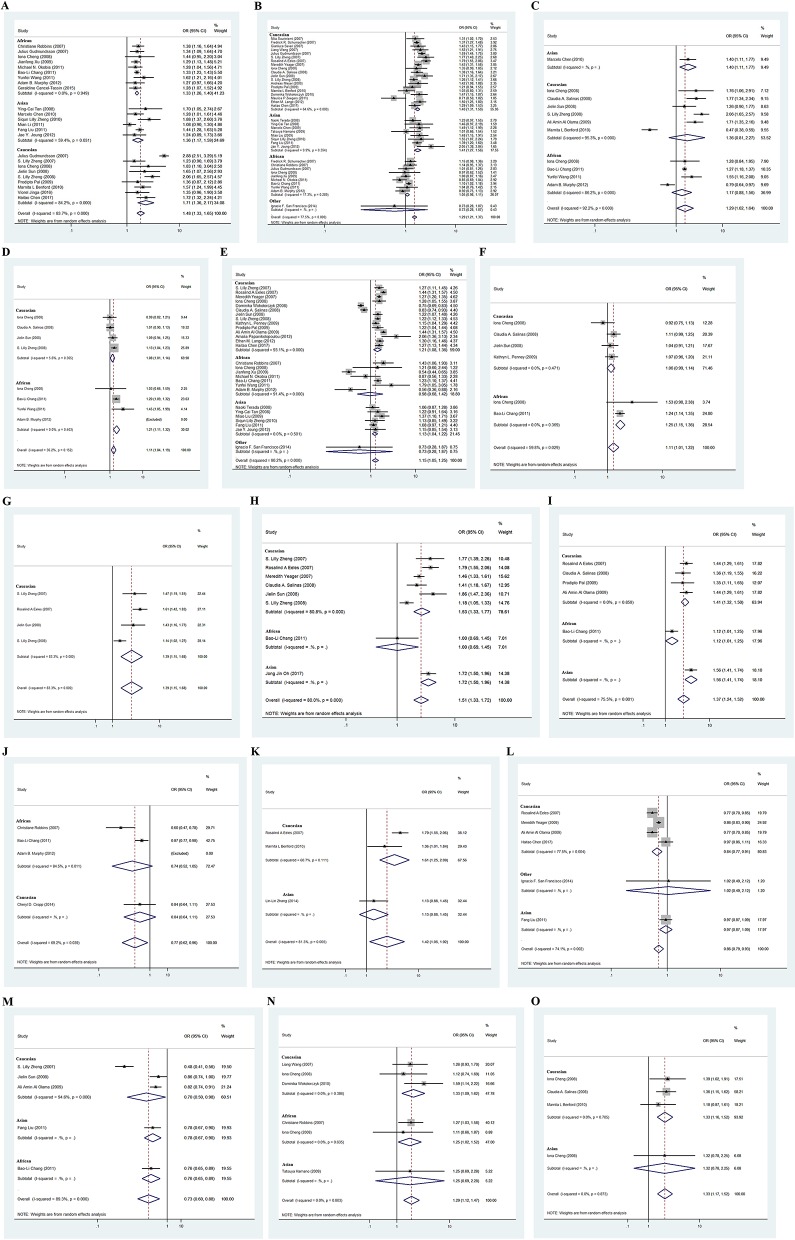
Forest plots for associations between selected variants in the 8q24 region and prostate cancer risk. Associations of rs16901979 **(A)**, rs1447295 **(B)**, rs6983561 **(C)**, rs7000448 **(D)**, rs6983267 **(E)**, rs13254738 **(F)**, rs7017300 **(G)**, rs7837688 **(H)**, rs1016343 **(I)**, rs7008482 **(J)**, rs4242384 **(K)**, rs620861 **(L)**, rs10086908 **(M)**, DG8S737 Allele−8 **(N)**, and rs10090154 **(O)** with prostate cancer risk.

Twenty-four studies were included (Table 1), and a significant association with prostate cancer risk was found (*p* = 1.08 × 10^−12^, random effect *OR* = 1.48, 95% *CI*: 1.33, 1.65; *Q* = 141.34, *p* = 0.00, *I*^2^ = 83.7%, Figure 2A). A similar pattern was observed for Africans (*p* = 1.26 × 10^−26^, random effect *OR* = 1.33, 95% *CI*: 1.26, 1.40; *Q* = 2.76, *p* = 0.949, *I*^2^ = 0.0%), Asians (*p* = 8.49 × 10^−5^, random effect *OR* = 1.36, 95% *CI*: 1.17, 1.59; *Q* = 12.31, *p* = 0.031, *I*^2^ = 59.4%) and Caucasians (*p* = 6.48 × 10^−6^, random effect *OR* = 1.72, 95% *CI*: 1.36, 2.17; *Q* = 50.60, *p* = 0.00, *I*^2^ = 84.2%). No publication bias was found in the eligible studies (Harbord's test *p* = 0.757, Table 2).

**Table 1:** Characteristics of the included articles.

**Study, year**	**Study design**	**Country/region**	**Ethnicity**	**Variant**	**Cases/controls**
Geraldine Cancel-Tassin, 2015 (Cancel-Tassin et al., 2015)	Population-based case–control study	France	African	rs16901979	489/534
Mian Li, 2011 (Li et al., 2011)	Case–control study	China	Asian	rs16901979	432/782
Maurice P Zeegers, 2011 (Zeegers et al., 2011)	Cohort Study	Netherlands	Caucasian	rs1447295	281/267
Marcelo Chen, 2010 (Chen et al., 2010)	Case–control study	China	Asian	rs16901979	331/335
				rs6983561	324/336
Prodipto Pal, 2009 (Pal et al., 2009)	Case–control study	USA	Caucasian	rs16901979	596/567
				rs1447295	
				rs6983267	
				rs4645959	
				rs1016343	
Marcelo Chen, 2009 (Chen et al., 2009)	Hospital-based case–control study	China	Asian	rs1447295	340/337
Andreas Meyer, 2009 (Meyer et al., 2009)	Hospital-based case–control study	Germany	Caucasian	rs1447295	486/462
				rs13281615	488/462
Iona Cheng, 2008 (Cheng et al., 2008)	Case–control study	USA	Caucasian	rs16901979	417/416
			African		89/87
				rs1447295	417/417
					89/89
				DG8S737	416/417
					89/89
				rs6983561	417/417
					88/89
				rs10090154	417/414
					89/88
				rs7000448	416/417
					89/89
				rs6983267	417/417
					89/89
				rs13254738	506/506
					89/88
Christiane Robbins, 2007 (Robbins et al., 2007)	Case–control study	USA	African	rs16901979	490/567
				rs1447295	
				DG8S737	
				rs6983267	
				rs7008482	
Miia Suuriniemi, 2007 (Suuriniemi et al., 2007)	Population-based case–control study	USA	Caucasian	rs1447295	582/538
Fredrick R. Schumacher, 2007 (Schumacher et al., 2007)	Nested case-control study	Multiple countries	Caucasian	rs1447295	5505/6270
			African		676/643
Julius Gudmundsson, 2007 (Gudmundsson et al., 2007)	Case–control study	Iceland	Caucasian	rs16901979	2663/5509
			African		373/372
			Caucasian	rs1447295	
			African		
Gianluca Severi, 2007 (Severi et al., 2007)	Case–control study	Australia	Caucasian	rs1447295	821/732
Dominika Wokołorczyk, 2008 (Wokolorczyk et al., 2008)	Case–control study	Poland	Caucasian	rs6983267	1910/1885
S. Lilly Zheng, 2007 (Zheng et al., 2007)	Case–control study	USA	Caucasian	rs16901979	1563/576
				rs1447295	
				rs6983267	
				rs4242382	
				rs7017300	
				rs7837688	
				rs4645959	
				rs10086908	
Jae Y. Joung, 2012 (Joung et al., 2012)	Hospital-based case–control study	Korea	Asian	rs16901979	194/169
				rs1447295	
				rs6983267	
Naoki Terada, 2008 (Terada et al., 2008)	Case–control study	Japanese	Asian	rs1447295	507/387
				rs6983267	
Michael N. Okobia, 2011 (Okobia et al., 2011)	Case–control study	Caribbean	African	rs16901979	338/426
				rs1447295	354/438
				rs6983267	343/426
Claudia A. Salinas, 2008 (Salinas et al., 2008)	Population-based case–control study	USA	Caucasian	rs1447295	1252/1233
				rs6983561	1264/1236
				rs10090154	1288/1250
				rs7000448	1262/1239
				rs6983267	1258/1238
				rs13254738	1256/1234
				rs7837688	1260/1241
				rs4645959	1261/1238
				rs1016343	1253/1233
				rs7837328	1258/1239
				rs16901966	1302/1260
				rs10505476	1256/1233
				rs7837328	1258/1239
				rs13281615	1254/1234
Marnita L Benford, 2010 (Benford et al., 2010)	Case–control study	USA	Caucasian	rs16901979	192/512
				rs1447295	189/523
				rs6983561	186/908
				rs10090154	189/505
				rs4242382	193/1167
				rs4242384	193/524
Siqun Lilly Zheng, 2010 (Zheng et al., 2010)	Population-based case–control study	China	Asian	rs16901979	283/145
				rs1447295	284/151
				rs6983267	282/152
Rosalind A Eeles, 2007 (Eeles et al., 2008)	Population-based case–control study	United Kingdom	Caucasian	rs1447295	1906/1934
				rs6983267	
				rs4242382	
				rs7017300	
				rs7837688	
				rs1016343	
				rs7837328	
				rs4242384	
				rs620861	
				rs16901966	
				rs7837328	
Jielin Sun, 2008 (Sun et al., 2008)	Population-based case–control study	USA	Caucasian	rs16901979	1625/560
				rs1447295	
				rs6983561	
				rs10090154	
				rs7000448	
				rs6983267	
				rs13254738	
				rs4242382	
				rs7017300	
				rs7837688	
				rs10086908	
Amalia Papanikolopoulou, 2011 (Papanikolopoulou et al., 2011)	Case–control study	Greece	Caucasian	rs6983267	86/99
Kathryn L. Penney, 2009 (Penney et al., 2009)	Case–control study	USA	Caucasian	rs6983267	1305/1402
				rs13254738	
Liang Wang, 2007 (Wang et al., 2007)	Case–control study	USA	Caucasian	rs1447295	1121/545
				DG8S737	
S. Lilly Zheng, 2008 (Zheng et al., 2008)	Population-based case–control study	Sweden	Caucasian	rs16901979	2893/1781
				rs1447295	
				rs6983561	
				rs10090154	
				rs7000448	
				rs6983267	
				rs4242382	
				rs7017300	
				rs7837688	
Ying-Cai Tan, 2008 (Tan et al., 2008)	Case–control study	India	Asian	rs16901979	153/227
				rs1447295	
				rs6983267	
Viorel Jinga, 2016 (Jinga et al., 2016)	Case–control study	Romania	Caucasian	rs16901979	955/1007
Cheryl D. Cropp, 2014 (Cropp et al., 2014)	Population-based case–control study	USA	Caucasian	rs7008482	522/510
Lin-Lin Zhang, 2014 (Zhang et al., 2014)	Case–control study	China	Asian	rs7837328	388/384
				rs4242384	
Ignacio F. San Francisco, 2014 (San Francisco et al., 2014)	Case–control study	Chile	Hispanic	rs1447295	83/21
				rs6983267	
				rs7837328	
				rs620861	
Adam B. Murphy, 2012 (Murphy et al., 2012)	Case–control study	Cameroon	African	rs16901979	308/469
				rs1447295	
				rs6983561	
				rs7000448	
				rs6983267	
				rs7008482	
Fang Liu, 2011 (Liu et al., 2011)	Case–control study	China	Asian	rs16901979	1108/1525
				rs1447295	
				rs6983267	
				rs620861	
				rs10086908	
Ethan M. Lange, 2012 (Lange et al., 2012)	Case–control study	USA	Caucasian	rs1447295	1176/1101
				rs6983267	
Bao-Li Chang, 2011 (Chang et al., 2011)	Case–control study	USA	African	rs16901979	2642/2584
				rs1447295	3167/3325
				rs6983561	2764/3255
				rs10090154	1683/1403
				rs7000448	1698/2329
				rs6983267	3666/2992
				rs13254738	2557/2277
				rs4242382	1289/1527
				rs7837688	636/330
				rs1016343	1975/1830
				rs7008482	2172/1760
				rs7837328	473/772
				rs10086908	861/876
				rs16901966	861/875
				rs10505476	473/744
				rs7837328	473/772
Yunfei Wang, 2011 (Wang et al., 2011)	Case–control study	USA	African	rs16901979	127/345
				rs1447295	
				rs6983561	
				rs10090154	
				rs7000448	
				rs6983267	
				rs4242382	
Tatsuya Hamano, 2010 (Hamano et al., 2010)	Case–control study	Japan	Asian	rs1447295	158/119
				DG8S737	
Dominika Wokołorczyk, 2010 (Wokolorczyk et al., 2010)	Hospital-based case–control study	Poland	Caucasian	rs1447295	690/602
				DG8S737	
Meredith Yeager, 2009 (Yeager et al., 2009)	Case–control study	USA	Caucasian	rs620861	10286/9135
				rs13281615	
Ali Amin Al Olama, 2009 (Al Olama et al., 2009)	Case–control study	United Kingdom	Caucasian	rs6983561	1906/1934
				rs10090154	
				rs6983267	
				rs1016343	
				rs620861	
				rs10086908	
Miao Liu, 2009 (Liu et al., 2009)	Case–control study	Japan	Asian	rs1447295	391/323
				rs6983267	
Jianfeng Xu, 2009 (Xu et al., 2009)	Case–control study	USA	African	rs16901979	868/878
				rs1447295	
				rs6983267	
Joke Beuten, 2009 (Beuten et al., 2009)	Cohort Study	USA	Caucasian	rs10505476	601/840
			hispanic		196/472
				rs7837328	
					
Meredith Yeager, 2007 (Yeager et al., 2007)	Cohort Study	USA	Caucasian	rs1447295	4296/4299
				rs6983267	
				rs7837688	
Jong Jin Oh, 2017 (Oh et al., 2017)	Hospital-based case–control study		Caucasian	rs1016343	1001/2641
				rs7837688	
Haitao Chen, 2018 (Chen et al., 2018)	Case–control study		Caucasian	rs6983267	779/1643
				rs620861	
				rs16901979	
				rs1447295	

**Table 2:** Details of genetic variants significantly associated with cancer risk in meta-analyses.

**Variants**	**Cancer risk**		**Initial study influence**		**Deviation from HWE**	***p*-value for publication bias**	***p*-value for small study bias**	**Genotype cancer risk**			
	**OR (95% CI)**	***p*-value**	**OR (95% CI)**	***p*-value**				***OR1* (95% CI)**	***p*-value**	***OR2* (95% CI)**	***p*-value**
rs16901979	1.48 (1.26–1.40)	1.08 × 10^−12^	1.49(1.33–1.66)	1.67 × 10^−12^	No	0.757	0.757	1.72(1.44–2.05)	1.97 × 10^−9^	1.36(1.15–1.61)	3.06 × 10^−4^
rs1447295	1.29 (1.21–1.37)	3.20 × 10^−14^	1.30 (1.21–1.39)	9.94 × 10^−15^	No	0.559	0.664	1.42(1.10–1.82)	0.006	1.31(1.18–1.45)	3.06 × 10^−7^
rs6983561	1.29 (1.02–1.64)	0.036	1.29 (1.00–1.66)	0.048	No	0.977	0.887	0.84(0.62–1.13)	0.242	1.54(1.29–1.83)	1.84 × 10^−6^
rs7000448	1.11(1.04–1.19)	0.003	1.11(1.03–1.20)	0.004	No	0.868	0.889	0.98(0.80–1.21)	0.867	1.04(0.90–1.20)	0.64
rs13254738	1.11(1.01–1.22)	0.026	1.13(1.04–1.23)	0.005	No	0.599	0.601	1.19(0.85–1.68)	0.312	1.04(0.94–1.16)	0.458
rs6983267	1.15(1.05–1.25)	0.003	1.14(1.04–1.25)	0.006	No	0.577	0.583	1.31(0.92–1.86)	0.134	1.05(0.5–1.22)	0.546
rs7017300	1.39(1.15–1.68)	0.001	1.37(1.08–1.75)	0.009	No	0.564	0.531				
rs7837688	1.51(1.33–1.72)	1.66 × 10^−10^	1.49(1.30–1.70)	1.20 × 10^−8^	No	0.921	0.816				
rs1016343	1.37(1.24–1.52)	8.25 × 10^−10^	1.36(1.20–1.54)	1.37 × 10^−6^	No	0.922	0.895				
rs7008482	0.77(0.62–0.96)	0.021	0.86(0.77–0.96)	0.008	No	0.549	0.533				
rs4242384	1.42(1.05–1.92)	0.022	1.22(1.01–1.48)	0.044	No	0.376	0.340				
rs620861	0.86(0.79–0.94)	3.57 × 10^−4^	0.89(0.81–0.97)	0.007	No	0.791	0.795				
rs10086908	0.73(0.60–0.88)	0.001	0.81(0.76–0.86)	1.66 × 10^−10^	No	0.339	0.428				
DG8S737−8 allele	1.29 (1.12–1.47)	3.06 × 10^−4^	1.29 (1.09–1.54)	0.004	No	0.592	0.648	0.83(0.29–2.38)	0.733	1.25(0.98–1.59)	0.068
rs10090154	1.33 (1.17–1.52)	2.04 × 10^−5^	1.33(1.16–1.52)	3.63 × 10^−5^	No	0.641	0.668	1.34(0.82–2.19)	0.245	1.40(1.2–1.62)	1.24 × 10^−5^

### rs1447295 C>A

Thirty-seven studies were included (Table 1), a significant association was found with the risk of prostate cancer (*p* = 3.20 × 10^−14^, random effect *OR* = 1.29, 95% *CI*: 1.21, 1.37; *Q* = 160.1, *p* = 0.00, *I*^2^ = 77.5%, Figure 2B). Significant association was also found for Asians (*p* = 2.08 × 10^−11^, random effect *OR* = 1.41, 95% *CI*: 1.27, 1.56; *Q* = 7.77, *p* = 0.354, *I*^2^ = 9.9%) and Caucasians (*p* = 2.52 × 10^−23^, random effect *OR* = 1.41, 95% *CI*: 1.31, 1.50; *Q* = 50.80, *p* = 0.00, *I*^2^ = 64.6%). However, no significant association was found for Africans (*p* = 0.168, random effect *OR* = 1.05, 95% *CI*: 0.98, 1.11; *Q* = 9.68, *p* = 0.289, *I*^2^ = 17.3%), No publication bias was found in the eligible studies (Harbord's test *p* = 0.587, Table 2).

### rs6983561 A>C

Eleven studies were included (Table 1), a significant association was found with the risk of prostate cancer (*p* = 0.036, random effect *OR* = 1.29, 95% *CI*: 1.02, 1.64; *Q* = 128.51, *p* = 0.00, *I*^2^ = 92.2%, Figure 2C). No significant association was found for Africans (*p* = 0.269, random effect *OR* = 1.17, 95% *CI*: 0.88, 1.56; *Q* = 21.67, *p* = 0.000, *I*^2^ = 86.2%) and Caucasians (*p* = 0.241, random effect *OR* = 1.36, 95% *CI*: 0.81, 2.27; *Q* = 105.31, *p* = 0.00, *I*^2^ = 95.3%). No publication bias was found in the eligible studies (Harbord's test *p* = 0.977, Table 2).

### rs7000448 C>T

Eight studies were included (Table 1), a significant association was found with the risk of prostate cancer (*p* = 0.003, random effect *OR* = 1.11, 95% *CI*: 1.04, 1.19; *Q* = 9.41, *p* = 0.152, *I*^2^ = 36.2%, Figure 2D). Further evaluation by ethnicity showed that significant association was found for Africans (*p* = 2.92 × 10^−5^, random effect *OR* = 1.21, 95% *CI*: 1.11, 1.32; *Q* = 1.82, *p* = 0.403, *I*^2^ = 0.0%) and Caucasians (*p* = 0.018, random effect *OR* = 1.08, 95% *CI*: 1.01, 1.14; *Q* = 3.18, *p* = 0.37, *I*^2^ = 5.6%). No publication bias was found in the eligible studies (Harbord's test *p* = 0.868, Table 2).

### rs6983267 T>G

Twenty-eight were included (Table 1), and a significant association with risk of prostate cancer was found (*p* = 0.003, random effect *OR* = 1.15, 95% *CI*: 1.05, 1.25; *Q* = 275.92, *p* = 0.00, *I*^2^ = 90.2%, Figure 2E). A similar pattern was observed for Asians (*p* = 0.003, random effect *OR* = 1.13, 95% *CI*: 1.04, 1.22; *Q* = 4.35, *p* = 0.501, *I*^2^ = 0.0%) and Caucasians (*p* = 0.001, random effect *OR* = 1.21, 95% *CI*: 1.08, 1.36; *Q* = 189.54, *p* = 0.00, *I*^2^ = 93.1%). No significant association was found for Africans (*p* = 0.269, random effect *OR* = 0.98, 95% *CI*: 0.68, 1.42; *Q* = 69.39, *p* = 0.000, *I*^2^ = 91.4%). No publication bias was found in the eligible studies (Harbord's test *p* = 0.577, Table 2).

### rs13254738 A>C

Six studies were included (Table 1), a significant association was found with the risk of prostate cancer (*p* = 0.026, random effect *OR* = 1.11, 95% *CI*: 1.01, 1.22; *Q* = 12.44, *p* = 0.029, *I*^2^ = 59.8%, Figure 2F). Significant association was found for Caucasians (*p* = 0.08, random effect *OR* = 1.06, 95% *CI*: 0.99, 1.14; *Q* = 2.52, *p* = 0.47, *I*^2^ = 0.0%). No publication bias was found in the eligible studies (Harbord's test *p* = 0.599, Table 2).

### rs7017300 A>C

Four studies were included, a significant association with prostate cancer risk was found (*p* = 0.001, random effect *OR* = 1.39, 95% *CI*: 1.15, 1.68; *Q* = 17.93, *p* = 0.000, *I*^2^ = 83.3%, Figure 2G). No publication bias was found in the eligible studies (Harbord's test *p* = 0.564, Table 2).

### rs7837688 G>T

Eight studies were included (Table 1), a significant association was found with the risk of prostate cancer (*p* = 1.66 × 10^−10^, random effect *OR* = 1.51, 95% *CI*: 1.33, 1.72; *Q* = 35.02, *p* = 0.000, *I*^2^ = 80.0%, Figure 2H). Significant association was also found for Caucasians (*p* = 3.64 × 10^−9^, random effect *OR* = 1.53, 95% *CI*: 1.33, 1.77; *Q* = 26.07, *p* = 0.000, *I*^2^ = 80.8%). No publication bias was found in the eligible studies (Harbord's test *p* = 0.921, Table 2).

### rs1016343 C>T

Six studies were included (Table 1), a significant association with risk of prostate cancer was found (*p* = 8.25 × 10^−10^, random effect *OR* = 1.37, 95% *CI*: 1.24, 1.52; *Q* = 20.42, *p* = 0.001, *I*^2^ = 75.5%, Figure 2I). Significant association was also found for Caucasians (*p* = 3.64 × 10^−9^, random effect *OR* = 1.41, 95% *CI*: 1.32, 1.50; *Q* = 0.76, *p* = 0.859, *I*^2^ = 0.0%). No publication bias was found in the eligible studies (Harbord's test *p* = 0.922, Table 2).

### rs7008482 G>T

Four studies were included (Table 1), a significant association was found with the risk of prostate cancer (*p* = 0.021, random effect *OR* = 0.77, 95% *CI*: 0.62, 0.96; *Q* = 6.49, *p* = 0.039, *I*^2^ = 69.2%, Figure 2J). No publication bias was found in the eligible studies (Harbord's test *p* = 0.549, Table 2).

### rs4242384 A>C

Three studies were included (Table 1), a significant association with prostate cancer risk was found (*p* = 0.022, random effect *OR* = 1.42, 95% *CI*: 1.02, 1.92; *Q* = 10.71, *p* = 0.005, *I*^2^ = 81.3%, Figure 2K). No publication bias was found in the eligible studies (Harbord's test *p* = 0.376, Table 2).

### rs620861 G>A

Six studies were included (Table 1), a significant association was found with the risk of prostate cancer (*p* = 3.57 × 10^−4^, random effect *OR* = 0.86, 95% *CI*: 0.79, 0.94; *Q* = 19.28, *p* = 0.002, *I*^2^ = 74.1%, Figure 2L). Significant association was also found for Caucasians (*p* = 3.64 × 10^−9^, random effect *OR* = 0.84, 95% *CI*: 0.77, 0.91; *Q* = 13.34, *p* = 0.004, *I*^2^ = 77.5%). No publication bias was found in the eligible studies (Harbord's test *p* = 0.791, Table 2).

### rs10086908 T>C

Five studies were included (Table 1), a significant association was found with the risk of prostate cancer (*p* = 3.57 × 10^−4^, random effect *OR* = 0.73, 95% *CI*: 0.60, 0.88; *Q* = 37.54, *p* = 0.000, *I*^2^ = 89.3%, Figure 2M). Significant association was also found for Caucasians (*p* = 0.036, random effect *OR* = 0.70, 95% *CI*: 0.50, 1.00; *Q* = 37.13, *p* = 0.004, *I*^2^ = 94.6%). No publication bias was found in the eligible studies (Harbord's test *p* = 0.339, Table 2).

### DG8S737 allele−8 absent>present

Five studies were included (Table 1), a significant association with risk of prostate cancer was found (*p* = 3.06 × 10^−4^, random effect *OR* = 1.29, 95% *CI*: 1.12, 1.47; *Q* = 2.32, *p* = 0.803, *I*^2^ = 0.0%, Figure 2N). A similar pattern was observed for Caucasians (*p* = 0.005, random effect *OR* = 1.33, 95% *CI*: 1.09, 1.62; *Q* = 1.91, *p* = 0.386, *I*^2^ = 0.0%). No publication bias was found in the eligible studies (Harbord's test *p* = 0.592, Table 2).

### rs10090154 C>T

Nine studies were included (Table 1), a significant association was found with the risk of prostate cancer (*p* = 2.04 × 10^−5^, random effect *OR* = 1.33, 95% *CI*: 1.17, 1.52; *Q* = 0.70, *p* = 0.873, *I*^2^ = 0.0%, Figure 2O). A similar pattern was observed for Caucasians (*p* = 3.63 × 10^−5^, random effect *OR* = 1.33, 95% *CI*: 1.16, 1.52; *Q* = 0.70, *p* = 0.705, *I*^2^ = 0.0%). No publication bias was found in the eligible studies (Harbord's test *p* = 0.641, Table 2).

## Genotype comparison

### rs16901979 C>A

Of the 24 studies, nine reported genotype information. The effects of genotype for AA vs. CC (*OR*1) and CA vs. CC (*OR*2) were calculated. Multivariate meta-analysis was conducted to estimate the pooled risk (Table 2). Individuals with the homozygous AA genotype (*p* = 3.86 × 10^−9^, random effect *OR*1 = 1.71, 95% *CI*: 1.43, 2.04; *Q* = 7.48, *p* = 0.486, *I*^2^ = 0.0%) and heterozygous CA genotype (*p* = 3.06 × 10^−4^, random effect *OR*2 = 1.36, 95% *CI*: 1.15, 1.61; *Q* = 14.29, *p* = 0.074, *I*^2^ = 44.0%) have increased risk of prostate cancer.

### rs1447295 C>A

Of the 38 studies, 19 reported genotype information. The effects of genotype for AA vs. CC (*OR*1) and CA vs. CC (*OR*2) were calculated for each study (Table 2). Individuals with the homozygous AA genotype (*p* = 0.006, random effect *OR*1 = 1.42, 95% *CI*: 1.10, 1.82; *Q* = 33.56, *p* = 0.010, *I*^2^ = 49.3%) and heterozygous CA genotype (*p* = 3.06 × 10^−7^, random effect *OR*2 = 1.31, 95% *CI*: 1.18, 1.45; *Q* = 38.05, *p* = 0.002, *I*^2^ = 55.3%) have increased risk of prostate cancer.

### rs6983561 A>C

Of the 11 studies, five reported genotype information. The genotype effects for CC vs. AA (*OR*1) and AC vs. AA (*OR*2) were calculated for each study (Table 2). There was a significantly increased risk of prostate cancer among individuals with heterozygous AC genotype (*p* = 1.84 × 10^−6^, random effect *OR*2 = 1.54, 95% *CI*: 1.29, 1.83; *Q* = 4.10, *p* = 0.393, *I*^2^ = 2.4%). However, no significant association was found among individuals with the homozygous CC genotype.

### rs10090154 C>T

Of the 9 studies, four reported genotype information. The effects of genotype for TT vs. CC (*OR*1) and CT vs. CC (*OR*2) were calculated for each study (Table 2). Individuals with heterozygous CT genotype (*p* = 1.24 × 10^−5^, random effect *OR*2 = 1.40, 95% *CI*: 1.20, 1.62; *Q* = 1.58, *p* = 0.663, *I*^2^ = 0.0%) have an increased risk of prostate cancer. However, no significant association was found among individuals with the homozygous TT genotype.

## Sensitivity analysis

Results of sensitivity analysis showed that the obtained results of 8q24 variants and risk of prostate cancer were robust statistically and no individual study affected the pooled OR significantly (Table 2).

## Discussion

To our knowledge, this study is the most comprehensive and largest evaluation of publications on associations between 8q24 variants and PCa risk. Preliminary meta-analyses mostly focused on the association between single or less SNPs with prostate cancer. From 46 eligible articles including 60,293 cases and 62,971 controls, we performed meta-analysis to evaluate associations between 15 variants in 8q24 region and PCa risk. Our study here provides an update of the previous reports. In addition, more variants were evaluated that have not been analyzed by meta-analyses previously.

Of the 21 variants located in 8q24, 15 were associated with prostate cancer risk significantly. Our primary analysis shows that, the rs16901979 (*p* = 1.08 × 10^−12^, *OR* = 1.48), rs1447295 (*p* = 4.51 × 10^−15^, *OR* = 1.29), rs6983561 (*p* = 0.036, *OR* = 1.29), rs7000448 (*p* = 0.003, *OR* = 1.11), rs6983267 (*p* = 0.003, *OR* = 1.15), rs13254738 (*p* = 0.026, *OR* = 1.11), rs7017300 (*p* = 0.001, *OR* = 1.39), rs7837688 (*p* = 1.66 × 10^−10^, *OR* = 1.51), rs1016343 (*p* = 8.25 × 10^−10^, *OR* = 1.37), rs7008482 (*p* = 0.021, *OR* = 0.77), rs4242384 (*p* = 0.022, *OR* = 1.42), rs620861 (*p* = 3.57 × 10^−4^, *OR* = 0.86), rs10086908 (*p* = 3.57 × 10^−4^, *OR* = 0.73), DG8S737 Allele-8 (*p* = 3.06 × 10^−4^, *OR* = 1.29), rs10090154 (*p* = 2.04 × 10^−5^, *OR* = 1.33) were significantly associated with PCa risk. In particular, both homozygous AA (*p* = 3.86 × 10^−9^, *OR*1 = 1.71) and heterozygous CA (*p* = 3.06 × 10^−4^, *OR*2 = 1.36) genotypes of rs16901979, as well as the AA (*p* = 0.005, *OR*1 = 1.41) and CA (*p* = 2.14 × 10^−8^, *OR*2 = 1.33) genotypes of rs1447295, were associated with PCa risk. Heterozygous AC genotype (*p* = 1.84 × 10^−7^, *OR*2 = 1.54) of rs6983561, CT genotype (*p* = 1.24 × 10^−5^, *OR*2 = 1.40) of rs10090154 were also found to be associated with the risk of PCa. Our findings were robust in regard to study design and sensitivity analyses according to several gene-variants-association studies and thousands of participants. No evidence of small study bias or publication bias was found.

The 8q24 region is dense with SNP (single-nucleotide-polymorphism) associated with risk for prostate, colorectal, breast cancer, et al. There are about five separated different cancer susceptibility loci specific for different cancers within the 8q24 “desert” (Huppi et al., 2012). Region 1, including rs16901979, rs13254738 and rs6983561, region 4, including rs7000448 and region 5, including rs1447295 specifically associated with the PCa risk, rs13281615 in region 2 is a breast-specific cancer susceptibility loci, rs10505477 and rs10808556 in a same block in region 3 were confirmed to be associated with colorectal cancer(Ghoussaini et al., 2008). Although the exact biological mechanisms underlying these associations with multiple cancers are confusing, these variants might affect tissue-specific enhancers of one or more genes involved in carcinogenesis. *FAM84B*, very closest to 8q24, is reported that, during prostate tumorigenesis and follows PCa progression, its expression increased (Wong et al., 2017). Another pseudogene of POU5F1P1/POU5F1B, located in 8q24.21 region, was also observed that levels of both the mRNA and protein increased in PCa (Kastler et al., 2010). Therefore, variants in 8q24 region themselves or with other variants might be responsible for the associations with prostate cancer.

Our study provides summary evidence that common 15 variants in the 8q24 region are associated with PCa risk. To explore the exact mechanisms of 8q24 variants involved in parthenogenesis of prostate cancer needs further functional studies.

## Author contributions

Data were extracted by YT and TY. SL, FZ, and JY analyzed the data. YQ and DM wrote the manuscript.

### Conflict of interest statement

The authors declare that the research was conducted in the absence of any commercial or financial relationships that could be construed as a potential conflict of interest.
